# Backbone and side-chain resonance assignments of the NISTmAb-scFv and antigen-binding study

**DOI:** 10.1007/s12104-022-10109-z

**Published:** 2022-09-09

**Authors:** Houman Ghasriani, Sara Ahmadi, Derek J. Hodgson, Yves Aubin

**Affiliations:** 1 Centre for Oncology, Radiopharmaceuticals and Research, Biologics and Radiotherapeutic Drugs Directorate, Health Canada, 251 Sir Frederick Banting Driveway, Ottawa, ON K1A 0K9 Canada; 2 Department of Chemistry, Carleton University, Ottawa, ON K1S 5B6 Canada

**Keywords:** Monoclonal antibody, Motavizumab, NISTmAb, NMR spectroscopy, Respiratory syncytial virus, Single-chain variable fragment

## Abstract

Monoclonal antibodies (mAbs) therapeutics are the largest and fastest growing class of biologic drugs, amongst which, the vast majority are immunoglobulin G1 (IgG1). Their antigen binding abilities are used for the treatment of immunologic diseases, cancer therapy, reversal of drug effects, and targeting viruses and bacteria. The high importance of therapeutic mAbs and their derivatives has called for the generation of well-characterized standards for method development and calibration. One such standard, the NISTmAb RM 8621 based on the antibody motavizumab, has been developed by the National Institute of Standards and Technologies (NIST) in the US. Here, we present the resonance assignment of the single chain variable fragment, NISTmAb-scFv, that was engineered by linking the variable domains of the heavy and light chains of the NISTmAb. Also, addition of a peptide, corresponding to the target antigen of motavizumab, to samples of NISTmAb-scFv has induced chemical shift perturbations on residues lining the antigen binding interface thereby indicating proper folding of the NISTmAb-scFv.

## Biological context

Monoclonal antibody therapeutics are the largest and fastest growing class of protein drugs for human use. Amongst these, single chain fragment variable (scFv) are small versions made by linking the variable regions of the heavy and light chains. ScFvs retain the antigen binding ability with essentially the same affinity as the parent mAb (Huston et al. 1988), and they allow the generation of libraries aimed at optimizing binding specificity and affinity, which is facilitated by the ability to produce scFv in *E. coli*. In addition, scFvs have been used as drugs, tools for radionuclide delivery on their own or incorporated in larger chimeric biologics for their antigen binding abilities (Ahmad et al. 2012; Monnier et al. 2013; Lu et al. 2020; Ferro Desideri et al. 2021).

The molecular recognition and the binding to the antigen are both functions that are associated with the variable fragment, “Fv”, of a monoclonal antibody (Lozano et al. 2012). As early as 1984, it was demonstrated that fragments from the heavy chain, which were recombinantly expressed in *E. coli*, had binding affinity for their epitopes (Cabilly et al. 1984), and in 1989 Ward and coworkers showed that a single immunoglobulin domain from the variable fragment (Fv) expressed in *E. coli*, had the ability to bind to its antigen (Ward et al. 1989). In the mid- and late 1980’s researchers were embarking on the idea of using inter-domain linkers for connecting two immunoglobulin domains from the heavy chain (V_H_) and the light chain (V_L_) of the variable region of a mAb, for recombinant production of a “single-chain variable fragment”, “scFv”, which could be used in therapeutic applications that required high specificity for target binding (Bird et al. 1988; Huston et al. 1988). These properties and their size make them interesting models to study the effects of therapeutic product excipients on the protein dynamics by NMR (Ghasriani et al. 2020). Product excipients are additives used to keep the protein active pharmaceutical ingredient stable during product manufacturing, product storage and delivery to patients.

We have focused on the variable fragment of the antigen-binding fragment of the NISTmAb, an IgG1κ monoclonal antibody derived from motavizumab (Schiel et al. 2018), and developed as a standard reference material for the characterization of therapeutic mAbs. Motavizumab was developed to target the fusion protein of the Respiratory Syncytial Virus (RSV-group). Respiratory syncytial virus (RSV-group) is a highly transmissible respiratory virus, which attacks the lower respiratory tract in children and adults and is responsible for the death of an estimate of 20,000 people in the US, and between 66,000 and 239,000 people worldwide every year (Thompson et al. 2003; Falsey et al. 2005; Nair et al. 2010; Lozano et al. 2012; Rha et al. 2020). The NISTmAb has been used in several multi-laboratory studies for assessing various analytical methods including NMR methods (Brinson et al. 2018) aimed for the characterization of therapeutic mAbs. The crystal structure of the antigen-binding fragment (F_ab_) of NISTmAb was determined (McLellan et al. 2011; Karageorgos et al. 2017). The crystal structure of NISTmAb bound to a 24-amino acid long peptide that corresponds to the epitope from RSV virus has also been determined (McLellan et al. 2010).

Here, we present the backbone and the side chain chemical shifts of the NISTmAb-scFv. In order to test whether the single-chain construct produced in *E. coli* adopted a biologically active conformation upon refolding, proton-nitrogen correlation maps were recorded in the presence and absence of the target peptide. We aim at using this single-chain variable fragment molecule as a first example of an immunoglobulin protein for in-depth studies of backbone dynamics and other motional properties in the presence of various therapeutic product excipients. Recently, a similar study on the 4-helix bundle filgrastim was conducted in our laboratory (Ghasriani et al. 2020).

## Methods and experiments

### Design of the scFv construct

The amino acid sequence of scFv was constructed from the sequence of the NIST-mAb reference material 8671 described by Formolo and coworkers (Formolo et al. 2015) (Karageorgos et al. 2017). Residues Q1 to S120 of the heavy chain (underlined) were linked to residues D1 to T108 of the light chain (*italicized*) using four (**GGGGS**) elements (Huston et al. 1988). The synthetic gene (Biobasic, Toronto, Canada) optimized for expression in *E. coli* was inserted in a modified pET15b vector containing ten histidines in the NdeI and BamH1 sites. After cleavage of the polyhistidine tag with thrombin, the resulting polypeptide product had 252 residues (26 kDa) with the following sequence: 
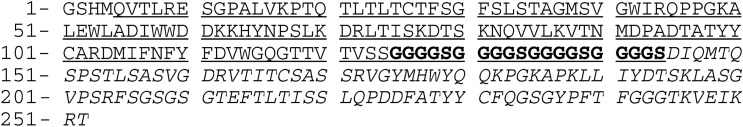
 where the first four extra residues resulted from the remainder of the thrombin cleavage site (GS) and the NdeI restriction enzyme site (HM) used for cloning. In order to match the numbering of residues in the NISTmAb-scFv with the NISTmAb numbering, Q1 in the NISTmAb heavy chain corresponds to Q4, and D1 in the NISTmAb light chain corresponds to D145.

### Expression and purification of scFv

Expression of labelled NISTmAb-scFv was carried out by incubating *E. coli* BL21(DE3) (Stratagene) harboring the pET15b10-NISTmAb-scFv plasmid in minimal media (M9) using 1 g/l ^13^C-glucose and 2 g/l ^15^N-ammonium chloride as sole source of carbon and nitrogen at 37 °C. Protein expression was induced by the addition of isopropyl thio-D-galactopyranoside (IPTG) at an OD_600_ of 0.8. Cells were harvested 3 h post-induction by centrifugation and frozen at − 80 °C until purification. Expression of labelled NISTmAb-scFv in *E. coli* resulted in the formation of inclusion bodies. Cell pellets corresponding to a 5 L culture were resuspended in 35 mL of buffer A (10 mM TrisHCl, 100 mM sodium phosphate, 6 M guanidine hydrochloride, 10 mM reduced glutathione, pH 8.0) and disrupted by sonication on ice using a 400 W Branson sonifier (ThermoFisher). After separation of cell debris, lysis was repeated once with 35 mL of buffer A and the supernatants were pooled and added to a slurry of Ni–NTA resin (Qiagen) (80 mL resin, 10 mL buffer A) and gently stirred at room temperature for 30 min before loading into a column. Refolding was accomplished under oxidative condition with a gradient of buffer A to B (Buffer B: 10 mM TrisHCl, 100 mM sodium phosphate, pH 8.0) over 20 column volumes. The column was then washed with three column volumes of Buffer B + 60 mM imidazole pH 8.0 to remove unspecific binding. The protein was eluted off the column with Buffer B + 250 mM imidazole (pH 8.0). The efficiency of the on-column refolding was such that the procedure was repeated several times (7 up to 10 times) to extract properly folded protein by re-equilibrating the column with buffer A followed by the above refolding-washing-elution protocol.

Prior to cleavage of the poly-histidine tag, the buffer was exchanged to 20 mM sodium phosphate (pH 6.0) by ultrafiltration. Cleavage was carried out at a protein concentration of 2 mg/mL using 1U of thrombin (Cytiva) per 100 µg of target protein at room temperature. While almost all starting material was cleaved after 30 min, the reaction was allowed to proceed overnight.

The reaction mixture was then purified on cation exchange chromatography using HiTrap SP FF columns (Cytiva) in 50 mM sodium phosphate buffer pH (6.0) with a 1 M sodium chloride salt gradient. The NISTmAb-scFv eluted at around 200 mM NaCl. Protein concentration was determined by using UV spectroscopy with the theoretical extinction coefficient 18,150 M^−1^ cm^−1^ (Swissprot). NMR samples contained 0.25 mM of the uniformly isotope-labeled ^13^C-^15^ N– or ^15^N-NISTmAb-scFv in 20 mM phosphate buffer at pH 6.0, and 5% ^2^H_2_O was used for field frequency lock. The sample temperature was kept at 313 K (40 °C).

### Peptide binding

The NMR titration of the peptide epitope with the NISTmAb-scFv was carried out with a 24-amino acid long polypeptide chain (NSELLSLINDMPLTNDQKKLMSNN), derived from the X-ray structure (PDBID 3ixt) epitope on the RSV virus fusion protein for motavizumab (McLellan et al. 2010).

The concentration of the stock peptide solution was 5.3 mM. A total of 10 μl of the stock solution was added to 550 μl of 1.65 mg ml^−1^ (~ 63 μM) protein solution, resulting in 95 μM peptide, and a final molar ratio of peptide-to-scFv of 1:0.65 (peptide being in excess). For recording of ^15^N-HSQC spectra of both the peptide-free and the peptide-bound scFv, the sample temperatures were kept at 308 K (35 °C).

### NMR experiments

Data were collected on Bruker NEO-600 and AVANCE IIIHD-700 MHz NMR spectrometers equipped with cryogenically cooled triple resonance inverse probes fitted with Z-axis gradients. For backbone resonance assignment, the standard double- and triple resonance experiments 2D-^15^N-HSQC (“*hsqcetfpf3gpsi*”)(Palmer et al. 1991; Kay et al. 1992; Grzesiek and Bax 1993b; Schleucher et al. 1994), 3D-HNCO (“*hncogp3d*”)(Grzesiek and Bax 1992; Schleucher et al. 1993; Kay et al. 1994), 3D-HN(CA)CO (“*hncacogp3d*”)(Clubb et al. 1992), 3D-CBCA(CO)NH (“*cbcaconhgp3d*”) (Grzesiek and Bax 1993a; Muhandiram and Kay 1994), 3D-HNCACB (“*hncacbgp3d*”) (Wittekind and Mueller 1993), 3D-HNCA (“*hncagp3d*”) (Grzesiek and Bax 1992), 3D-HN(CO)CA (“*hncocagp3d*”) (Grzesiek and Bax 1992), were recorded. For assignment of the side chain ^1^H and ^13^C chemical shifts, the double- and triple resonance experiments 2D-^13^C-HSQC (“*hsqcctetgpsisp*”) (Palmer et al. 1991; Vuister and Bax 1992), 3D-H(CC)(CO)NH (“*hccconhgp3d2*”) and 3D-(H)CC(CO)NH (“*hccconhgp3d3*”) (Montelione et al. 1992; Clowes et al. 1993; Grzesiek et al. 1993; Logan et al. 1993; Lyons and Montelione 1993; Carlomagno et al. 1996), 3D-HA(CO)NH (“*haconhgpwg3d*”) (Grzesiek and Bax 1993a; Muhandiram and Kay 1994), 3D-HANH (“*hanhgpwg3d*”) (Kuboniwa et al. 1994; Weisemann et al. 1994), 3D-HBHA(CO)NH (“*hbhaconhgp3d*”) (Grzesiek and Bax 1993a; Muhandiram and Kay 1994), 3D-HBHANH (“*hbhanhgpwg3d*”), 3D-(H)N(CA)NNH (“*hncannhgpwg3d*”) (Weisemann et al. 1993), 3D-CCHTOCSY (“*hcchdigp3d2*”) (Kay et al. 1993), 3D-HCCH-COSY (“*hcchcogp3d*”) (Kay et al. 1993), 3D-HCCH-TOCSY (“*hcchdigp3d*”) (Kay et al. 1993), 3D-^13^C-NOESY-HSQC (“*noesyhsqcetgpsi3d*”)(Palmer et al. 1991; Kay et al. 1992; Schleucher et al. 1994), and 3D-^15^N-NOESY-HSQC (“*noesyhsqcf3gpsi3d*”)(Palmer et al. 1991; Kay et al. 1992; Schleucher et al. 1994) were recorded. For assignment of aromatic side chain H^δ^ and H^ε^, as well as H^δ1^_Trp_, the Yamazaki experiments, 2D-CB(CGCD)HD (“*hbcbcgcdhdgp*”) and 2D-CB(CGCDCE)HE (“*hbcbcgcdcehegp*”)(Yamazaki et al. 1993), were recorded. The names of the pulse programs for experiments selected from the standard Bruker library are written in brackets.

### Data analysis and the assignment

All NMR data were processed using NMRPipe software (Delaglio et al. 1995). NMRFAM-Sparky(Goddard and Kneller; Lee et al. 2015) was employed for spectral visualization and spectral analysis. Automatic assignment routine PINE-Sparky(Lee et al. 2019) was invoked for probabilistic assignment of the backbone amide ^1^H, ^15^N, and backbone carbonyl ^13^C chemical shifts. Assignment of side chain resonances was done through a semi-automatic approach by initial engagement of PINE-Sparky, followed by implementation of an “inspection–verification” strategy, where PINE result for each assignment was either accepted or rejected based on a holistic approach that included inspection of complementary NOESY data.

## Extent of assignments and data deposition

**Fig. 1:**
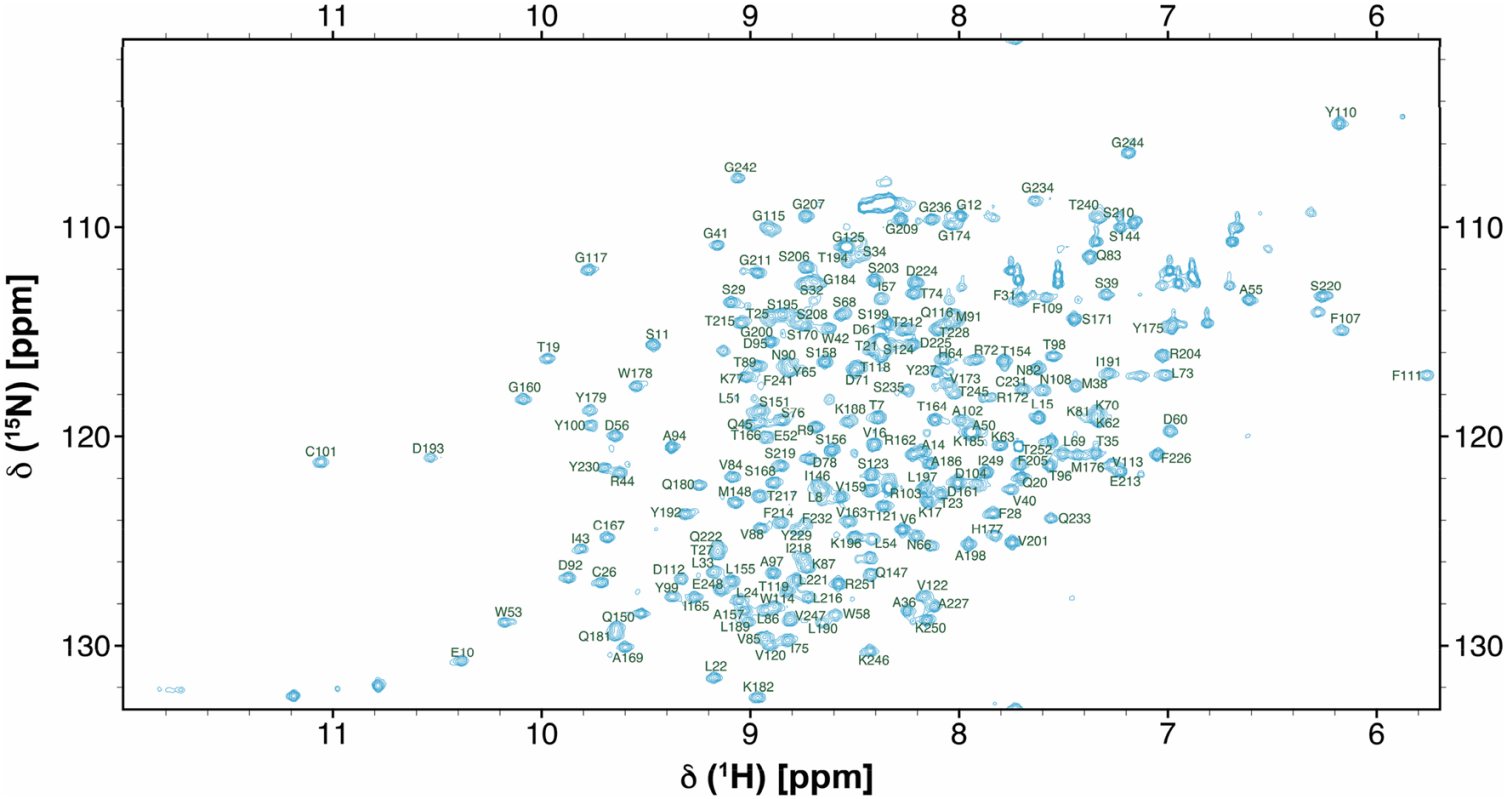
Two-dimensional 15N-HSQC spectrum of 13C-15N-NISTmAb-scFv at 700 MHz recorded at 35 °C. Peaks are assigned according to the residue number of our construct (see text)

The scFv construct was comprised of 252 residues, which included the 20-amino acid long linker. All but 19 backbone ^1^H–^15^N resonances in the ^15^N-HSQC spectrum were successfully assigned (91% completeness, Fig. 1). The number of missing and unassigned resonances for backbone ^13^C^α^ and ^13^CO were 16 and 42, respectively. These numbers correspond to 94% and 83% assignment completeness for these two heavy backbone atoms, respectively. All the missing resonances reside exclusively in the loops and in the random coil regions (Met^148^-Ser^153^, and Gly^242^-Gly^244^). Of the total of 123 methyl groups from 84 methyl-containing residues in the ^13^C-HSQC spectrum (Ala^β^, Ile^γ^, Ile^δ^, Leu^δ^, Met^ε^, Thr^γ^, and Val^γ^), 118 ^13^C-^1^H_3_ pairs were assigned (96% completeness). In total, 207 of 232 residues had their side chains fully, or partially, assigned (89% completeness). In all, the assigned backbone and side chain chemical shifts comprising of ^1^H (1113), ^13^C (789), and ^15^ N (215) were deposited into BMRB database (access code: 51094).

A number of residues, namely Ala^36^, Ile^43^, Arg^44^, Leu^51^, Asp^60^, Leu^73^, Leu^86^, Cys^101^, Ala^102^, Asn^108^, Tyr^110^, Phe^111^, Pro^187^, Gly^207^, Leu^216^, Ser^220^, and Cys^231^, displayed unusual chemical shifts that we attributed to the shielding effects afforded by close packing to aromatic rings based on analysis of the X-ray structure (PDBID 5k8a).

Three other types of unusual shifts (deshielding effects) were (a) due to either intra-residual side chain to main chain hydrogen bonding (Eswar and Ramakrishnan 2000) (the case of amide proton of Glu^10^), (b) due to ring current(Wieloch 1978) from a protonated histidine imidazole ring reaching across from the neighboring strand (the case of nitrogen atom of Asp^193^), or (c) due to a potential, but unusual, hydrogen bond between methyl protons and backbone carbonyl oxygen of residue i-4,(Yesselman et al. 2015) resulting from helical conformation in the middle of a loop (the case of methyl protons of Leu^73^). The extreme shift for backbone nitrogen atom of Asp^193^ can be attributed to a deshielding effect caused by ring current from protonated histidine imidazole ring of His^177^ (Wieloch 1978). The anomalous chemical shift for backbone nitrogen atom of Gly^207^ which displayed a down-field shift compared to average glycine chemical shift, can be attributed to side-chain to backbone H-bonding to the adjacent Ser^206^ (Vijayakumar et al. 1999; Eswar and Ramakrishnan 2000). Two particularly challenging regions for assignment, were the final stretch of beta stand that follows the binding loop (Ser^235^-Gly^244^), and the two short loops that are in close proximity of each other from heavy and light chains (Gln^45^-Lys^49^, and Lys^182^-Ala^186^). These regions account for 15 of the unassigned ^13^CO, 6 of the unassigned ^13^C^α^, and 3 of the unassigned ^15^N–^1^H backbone resonances.

The signals from six methionine methyl groups were well-dispersed in the ^13^C-HSQC spectrum, such that the ^13^C^ε^ and ^1^H^ε^ chemical shifts could be readily identified using a combination of ^13^C-noesy and ^15^N-noesy spectra.

### Truncation or crystal effect

**Fig. 2:**
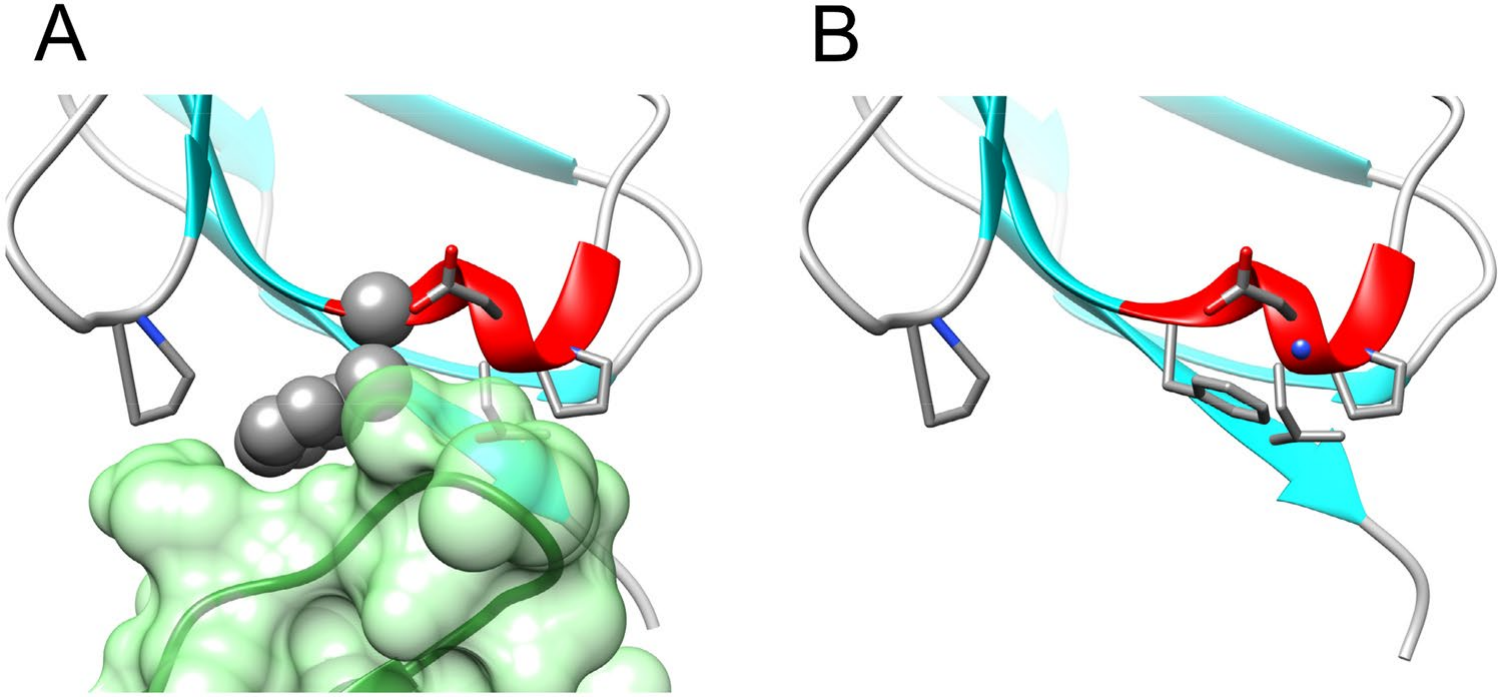
**a** Phe82 (represented in CPK) in the NISTmAb-Fab domain adopts a rotamer that is locked due the proximity of the constant light (CL) domain (green surface).**b** In the NISTmAb-scFv, the absence of the CL domain allows Phe226 (stick) to adopt a rotamer in NISTmAb-scFv that induces a shielding effect on Asp224 amide resonance (blue sphere)

An interesting finding that emerged while we were examining the 3D crystal structure for plausible explanation of the anomalous and extreme chemical shift of backbone amide nitrogen of Asp^224^, was the positioning of aromatic ring of Phe^226^ (Fig. 2a). As evidence, we later found NOE signals, which confirmed that the orientation of the phenylalanine ring in our case is in opposition to the one in the crystal structure of NISTmAb-F_ab_. For example, we observed NOESY cross peaks from both Pro^223^ and Ile^249^ to the aromatic ring of Phe^226^. Since the loops of the constant fragment in the F_ab_ fragment (*residues Glu*^*164.L*^*, Gln*^*165.L*^*, and Asp*^*166.L*^) would physically restrict the rotation of the aromatic ring of Phe^226^ (*Phe*^*82.L*^), we speculate that the rotation about the Cα-Cβ bond of Phe^226^ in NISTmAb-scFv is made possible by the absence of constant domains C_H_1 and C_L_. (Fig. 2b). Of course, this does not take into account any “crystal effects” that would have contributed to a dense/strained packing of the loop against the aromatic ring in the full F_ab_.

### Peptide binding

**Fig. 3:**
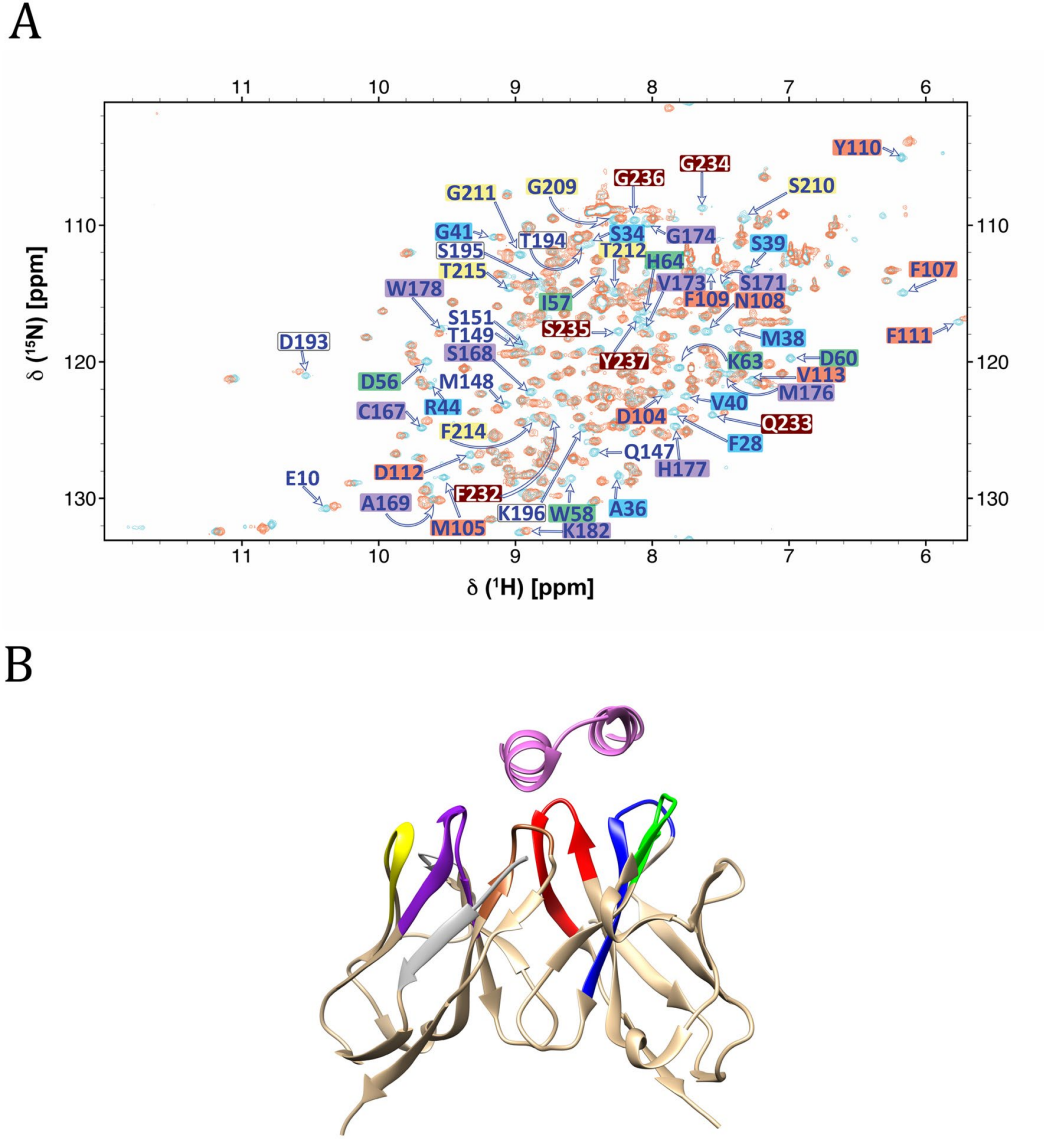
**a** Overlay of 2D-15N-HSQC spectra of 13C-15N-NISTmAb-scFv free (blue) and peptide-bound (red) at 700 MHz recorded at 35 °C. Residues showing chemical shift perturbation upon peptide binding are indicated with a color code (red, green, blue, brown, yellow, grey-white and purple. **b** Ribbon diagram of the variable domains (VH and CL) of the NISTmAb-Fab X-ray structure (PDBID: 3IXT). Residues experiencing CSP depicted in panel A are mapped on the structure using the same colors

In order to verify that refolding of the NISTmAb-scFv polypeptide led to a biologically active conformation similar to the NISTmAb-Fab fragment, we carried out a simple peptide binding experiment. Two dimensional ^15^N-HSQC spectra were recorded for peptide-free and peptide-bound ^15^N-NISTmAb-scFv (Fig. 3). The chemical shift perturbations of backbone amide pairs were largest for residues involved in the peptide-binding site, consistent with the residues in close proximity with the peptide in the crystal structure of the complex (PDBID 3IXT) (McLellan et al. 2010). This observation indicates that the NISTmAb-scFv has folded into a biologically active conformation.

## Data Availability

Chemical shifts and Bruker raw data *ser* files were deposited in the BMRB data bank with entry number 51094.

## Abbreviations

mAb: Monoclonal antibody
scFv: single-chain variable fragment
VH: heavy chain variable domain
VL: light chain variable domain
Fab: antigen-binding fragment
NISTmAb: National Institute of Standards and Technology monoclonal antibody
RSV: Respiratory syncitial virus
